# Nardosinane-Type Sesquiterpenoids from the Formosan Soft Coral *Paralemnalia thyrsoides*

**DOI:** 10.3390/md9091543

**Published:** 2011-09-16

**Authors:** Chiung-Yao Huang, Jui-Hsin Su, Bo-Wei Chen, Zhi-Hong Wen, Chi-Hsin Hsu, Chang-Feng Dai, Jyh-Horng Sheu, Ping-Jyun Sung

**Affiliations:** 1 Department of Marine Biotechnology and Resources, National Sun Yat-sen University, Kaohsiung 804, Taiwan; E-Mails: betty8575@yahoo.com.tw (C.-Y.H.); x2219@nmmba.gov.tw (J.-H.S.); a6152761@yahoo.com.tw (B.-W.C.); wzh@mail.nsysu.edu.tw (Z.-H.W.); hsuch@mail.nsysu.edu.tw (C.-H.H.); 2 National Museum of Marine Biology & Aquarium, Pingtung 944, Taiwan; 3 Institute of Marine Biotechnology, National Dong Hwa University, Pingtung 944, Taiwan; 4 Institute of Oceanography, National Taiwan University, Taipei 112, Taiwan; E-Mail: corallab@ntu.edu.tw; 5 Division of Marine Biotechnology, Asia-Pacific Ocean Research Center, National Sun Yat-sen University, Kaohsiung 804, Taiwan

**Keywords:** soft coral, *Paralemnalia thyrsoides*, nardosinane, neuroprotective activity

## Abstract

Five new nardosinane-type sesquiterpenoids, paralemnolins Q–U (**1**–**5**), along with three known compounds (**6**–**8**), were isolated from the Formosan soft coral *Paralemnalia thyrsoides*. The structures of new metabolites were elucidated on the basis of extensive spectroscopic methods, and the absolute configuration of **1** was determined by the application of Mosher’s method on **1**. Among these metabolites, **1** and **3** are rarely found nardosinane-type sesquiterpenoids, possessing novel polycyclic structures. Compounds **1**, **3**, **6** and **7** were found to possess neuroprotective activity.

## 1. Introduction

**Chart 1:**
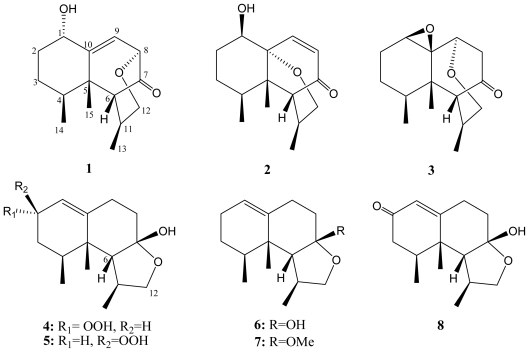
Structures of metabolites **1**–**8**.

Soft corals of the genus *Paralemnalia* [1–7] and *Lemnalia* [8–12] have been found to be rich sources of sesquiterpenoids of nardosinane [1–3,9,12,13], neolemnane [3,4,10,11] and africanane-type [3,8] compounds and norsesquiterpenoids of nornardosinane-type [3,5,6,9,10] compounds. Our previous study on the secondary metabolites of a Taiwanese soft coral *Paralemnalia thyrsoides*, collected from the coast of Green Island, has resulted in the isolation of two norsesquiterpenoids [6], ten sesquiterpenoids [4,6,7] and three novel sesquiterpenoids derived from a nardosinane precursor [7]. In our continuing search for new and bioactive metabolites from Formosan soft corals, *Paralemnalia thyrsoides* collected from Orchid Island were chemically investigated for the first time, and the investigation has resulted in the isolation of five new nardosinane-type sesquiterpeneoids, paralemnolins Q–U (**1**–**5**), along with three known compounds 2-deoxylemnacarnol (**6**) [2], 2-deoxy-7-*O*-methyllemnacarnol (**7**) [14] and 2-oxolemnacarnol (**8**) [15] (Chart 1). The structures of sesquiterpenoids **1**–**5** were elucidated by spectroscopic analysis and the absolute configurations were established by application of modified Mosher’s method on **1** [16]. The inhibitory activity of compounds **1**–**8** against three human cancer cell lines was investigated, however, none of these metabolites was found to possess useful cytotoxicity. Furthermore, a study of the neuroprotective effect of these metabolites revealed that **1**, **3**, **6** and **7**, in particular **6**, could reduce 6-OHDA (6-hydroxydopamine)-induced neurotoxicity in neuroblastoma SH-SY5Y cells.

## 2. Results and Discussion

**Figure 1:**
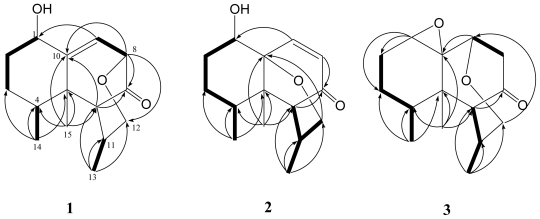
Selected ^1^H–^1^H COSY (**—**) and HMBC (→) correlations of **1**–**3**.

Paralemnolin Q (**1**) was obtained as a white powder. The HRESIMS (*m/z* 273.1468 [M + Na]^+^) of **1** established the molecular formula C_15_H_22_O_3_, appropriate for five degrees of unsaturation, and its IR spectrum revealed the presence of carbonyl (1735 cm^−1^) and hydroxy (3429 cm^−1^) groups. The ^13^C NMR and DEPT (Table 1) spectroscopic data showed signals of three methyls, three sp^3^ methylenes (including one oxymethylene appearing at δ_C_ 69.5), five sp^3^ methines, one sp^2^ methine, one sp^3^ and two sp^2^ quaternary carbons (including one carbonyl carbon appearing at δ_C_ 211.6). The above data accounted for two of the five degrees of unsaturation, indicating a tricyclic structure for **1**. From the COSY spectrum measured in CDCl_3_, it was possible to establish four proton sequences from H-1 to H_2_-3, H-4 to H_3_-14, H-8 to H-9 and H-11 to H_3_-13 (Figure 1). Key HMBC correlations of H-6 to C-7; H-8 to C-7 and C-10; H-9 to C-1; H_3_-13 to C-6, C-11 and C-12; H_3_-14 to C-3, C-4 and C-5; and H_3_-15 to C-4, C-5, C-6 and C-10, permitted the connection of the carbon skeleton. Furthermore, the HMBC cross-peak from H-8 to C-12 suggested that C-8 and C-12 were linked through an oxygen to form a tetrahydropyran ring. On the basis of the above analysis, the gross planar structure of **1** was established.

**Figure 2:**
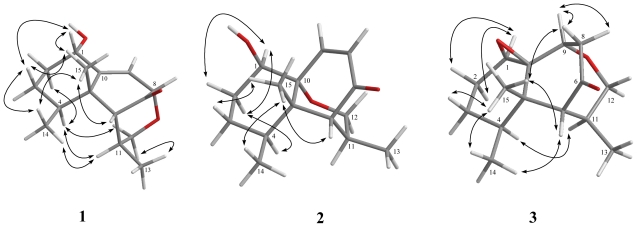
Key NOESY correlations for **1**–**3**.

**Figure 3:**
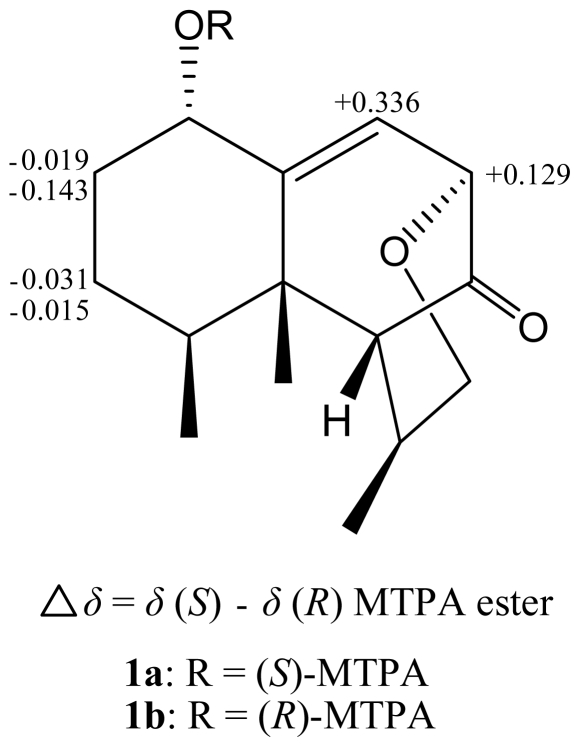
^1^H NMR chemical shift differences of MTPA esters of **1**.

The relative configuration of **1** was elucidated by the analysis of NOE correlations, as shown in Figure 2. It was found that H_3_-15 (δ_H_ 0.98, s) showed NOE interactions with H-1 (δ_H_ 4.22, dd, *J* = 12.0, 4.8 Hz), H-6 (δ_H_ 2.10, brs), and H_3_-14 (δ_H_ 0.79, d, *J* = 6.4 Hz); therefore, assuming the β-orientation of H_3_-15, all of H-1, H-6, and H_3_-14 should also be positioned on the β face. Furthermore, H-4 (δ_H_ 2.02, ddq, *J* = 12.0, 4.0, 6.4 Hz) exhibited NOE correlations with H-11 (δ_H_ 2.39, ddq, *J* =3.6, 3.2, 7.2 Hz) and one proton of H_2_-12 (δ_H_ 4.18, dd, *J* = 12.0, 3.2 Hz), revealing the α-orientation of H-11, and the β-orientations of H-8 and H_3_-13. On the basis of the above findings and other detailed NOE correlations (Figure 2), the relative structure of **1** was determined. In order to resolve the absolute structure of **1**, we determined the absolute configuration at C-1 using a modified Mosher’s method [15]. The (*S*)- and (*R*)-MTPA esters of **1** (**1a** and **1b**, respectively) were prepared using the corresponding *R*-(−)- and *S*-(+)-α-methoxy-α-(trifluoromethyl)phenylacetyl chlorides, respectively. The determination of the chemical shift differences (δ*_S_* – δ*_R_*) for the protons neighboring C-1 led to the assignment of the *S* configuration at C-1 of **1** (Figure 3). Thus, the absolute configuration of **1** has been determined.

The HRESIMS spectrum of paralemnolin R (**2**) showed a molecular formula of C_15_H_22_O_3_, the same as that of **1**. The NMR data revealed the presence of an α,β-unsaturated ketone (δ_C_ 201.8 C), and one trisubstituted double bond (δ_C_ 143.6 CH, 133.9 CH). The above functionalities account for two of the five degrees of unsaturation, suggesting a tricyclic structure in **2**. ^1^H–^1^H COSY and HMBC spectra (Figure 1) further revealed that **2** possesses an α,β-unsaturated ketone at C-7(C=O), C-8 and C-9. Furthermore, the HMBC cross-peak from H-12 to C-10 suggested that C-10 and C-12 are linked through an oxygen. On the basis of the above observations, and by the assistance of additional 2D NMR (^1^H–^1^H COSY and HMBC) correlations, it was possible to establish the planar structure of **2** as illustrated in Figure 1. The relative configurations of the six chiral centers at C-1, C-4, C-5, C-6, C-10 and C-11 in **2** were further determined on the basis of NOE correlations (Figure 2). It was found that H_3_-15 showed NOE interactions with both H_3_-14 and H-6, while H-4 was NOE correlated with H-11. Therefore, H-6, H_3_-14 and H_3_-15 were positioned on the same β-face, and in contrast, H-4 and H-11 should be placed on the α-face. Moreover, one of the methylene protons at C-2 (δ_H_ 2.18, dddd, *J* = 15.0, 15.0, 5.0, 3.5 Hz) exhibited NOE correlations with H-4 and was assigned as H-2α. The NOE correlation observed between H-2α and H-1 reflected the β-orientation of hydroxy group. Further NOE analysis revealed that **2** possesses the same configurations at C-4, C-5, C-10, and C-11, as in known compounds flavalins B-D [12]. Thus, the structure of **2** was fully established.

The HRESIMS of paralemnolin S (**3**) showed that it possesses the molecular formula C_15_H_22_O_3_ (*m/z* 251.1634 [M + H]^+^). The IR spectrum of **3** showed the absorption of a carbonyl group (1719 cm^−1^). The NMR data showed the presence of one trisubstituted epoxide (δ_H_ 3.31, 1H, brs; δ_C_ 62.8, C and 59.0, CH), and one ketone (δ_C_ 210.6, C). The above functionalities and ^1^H and ^13^C NMR spectroscopic data (Tables 1 and 2) showed a polycyclic structure in **3**. On the basis of the above results and by the assistance of ^1^H–^1^H COSY and HMBC spectroscopic analyses (Figure 1), the molecular framework of **3** could be established. This metabolite was found to be a rare nardosinane containing an oxacycloheptane. The 4S*, 5S*, 11R* configurations of **3** were revealed from the similar NOE interactions (Figure 2) as in **1** and **2**. Moreover, by NOESY spectrum (Figure 2), it was found that the α-oriented H-1 showed NOE interactions with H_2_-2, but not with H_3_-15, indicating the α-orientation of H-1. Furthermore, the NOE correlation observed between one proton (δ_H_ 2.80, dd, *J* =19.2, 2.8 Hz) of H_2_-8 with H_3_-15 and H-9 reflected the β-orientation of H-9. From the above evidences and the other NOE correlations (Figure 3), the structure of **3** was determined.

**Figure 4:**
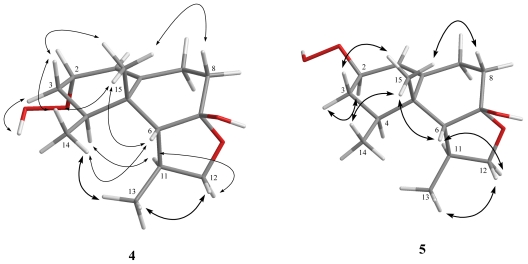
Key NOE correlations of **4** and **5**.

The HRESIMS of paralemnolin T (**4**) showed that it possesses the molecular formula C_15_H_24_O_4_ (*m/z* 269.1742 [M + H]^+^). The IR spectrum of **4** showed the absorption of a hydroxy group (3361 cm^−1^). Comparison of the ^1^H and ^13^C NMR spectroscopic data (Tables 1 and 2) of compounds **4** and **8** [15] suggested that the structure of **4** should be very similar to that of **8**, with the exception of signals assigned to C-2, where a ketone (δ_C_ 198.0, C) in **8** was replaced by one hydroperoxy-bearing methine (δ_H_ 4.37, 1H, t, *J* = 4.0 Hz, δ_C_ 77.8, CH) in **4**. Furthermore, H_3_-15 was found to show NOE correlations with H-6, H_3_-14 which further correlates with H_3_-13, and one proton of H_2_-3 (δ_H_ 1.57, m), and the other proton of H_2_-3 (δ_H_ 1.93, m) showed an NOE correlation with hydroperoxy proton (δ_H_ 7.83, brs), suggesting that H-2, H-6, H_3_-13, H_3_-14 and H_3_-15 should be placed on the same β-face and in contrast, hydroperoxy group should be positioned on the α-face. Further analysis of other NOE correlations revealed that **4** possesses the same relative configurations at C-4, C-5, C-6, C-7 and C-11 as those of **8** (Figure 4). Based on the above results, the structure of **4** was established.

Paralemnolin U (**5**) was isolated as a white solid. Its HRESIMS exhibited a [M + H]^+^ ion peak at 269.1746 *m*/*z*, establishing a molecular formula of C_15_H_24_O_4_. By 2D NMR spectroscope data, including COSY, HMQC, and HMBC, compound **5** was shown to possess the same molecular framework as that of **4**. Furthermore, the NMR data of **5** were very similar to those of **4**, suggesting that **5** is an isomer of **4**. By NOESY spectrum (Figure 2), it was found that the β-oriented H_3_-15 showed NOE correlations with one proton of H_2_-3 (δ_H_ 1.56, m) and the other proton of H_2_-3 (δ_H_ 1.84, m) showed NOE correlation with H-2, indicating the β-orientation of the hydroperoxy group. On the basis of the above findings and other NOE correlations (Figure 4), **5** was revealed to be the C-2 epimer of **4**.

**Figure 5:**
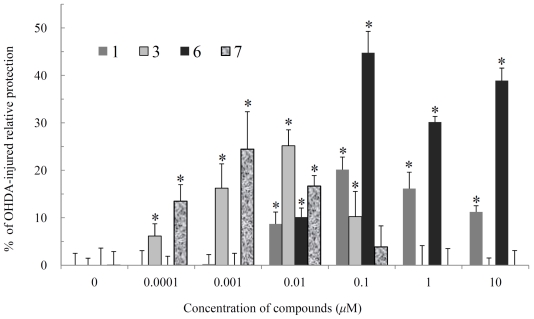
The neuroprotective effects of **1**, **3**, **6** and **7** on 6-OHDA-induced neurotoxicity in human neuroblastoma SH-SY5Y cells. SH-SY5Y cells were pre-incubated for 1 h with the indicated concentration of test compound and then stimulated with 6-OHDA (20 μM) or vehicle. Relative neuroprotection of control (without the treatment of 6-OHDA and compound) and 6-OHDA-treated alone group were taken to be 100% and 0%, respectively. The experiment was repeated three times. * Significantly different from the 6-OHDA-treated alone group (*P* < 0.05).

In order to explore the biological activities of the isolated compounds, cytotoxicity of these compounds against the proliferation of a limited panel of cancer cell lines, including mouse melanoma (B16), human epithelial carcinoma (HeLa), human hepatoma carcinoma (HepG2) cell lines, was evaluated. The results showed that all of the compounds were not cytotoxic toward the above cancer cells (IC_50_’s > 20 μg/mL). Furthermore, the neuroprotective assay of **1**–**8** using 6-OHDA-induced neurotoxicity in neuroblastoma SHSY5Y, a human dopaminergic neuron often used for study of Parkinson’s disease [17], was performed by a method reported previously [18]. It was observed that the cytotoxicity of 6-OHDA on SH-SY5Y cells could be reduced by pretreatment with **1**, **3**, **6** and **7** at various concentrations. The relative neuroprotective activities of **1** at 10^−2^, 10^−1^, 1 and 10 μM were 8.7 ± 2.5, 20.2 ± 14.0, 16.2 ± 3.5 and 11.2 ± 1.3%, **3** at 10^−4^, 10^−3^, 10^−2^ and 10^−1^ μM were 6.1 ± 2.6, 16.2 ± 5.1, 25.2 ± 3.4 and 10.2 ± 5.3%, **6** at 10^−2^, 10^−1^, 1 and 10 μM were 10.1 ± 1.9, 44.8 ± 4.5, 30.2 ± 1.2 and 38.9 ± 2.7%, and **7** at 10^−4^, 10^−3^ and 10^−2^ μM were 13.5 ± 3.5, 24.5 ± 7.9 and 16.7 ± 2.2%, respectively (Figure 5). From the neurological activity results, we suggest that further investigation of **1**, **3**, **6** and **7** for their therapeutic potential against neurodegenerative diseases is worthwhile.

## 3. Experimental Section

### 3.1. General Experimental Procedures

Melting points were determined using a Fisher-Johns melting point apparatus. Optical rotations were measured on a JASCO P-1020 polarimeter. Ultraviolet spectrum was recorded on a JASCO V-650 spectrophotometer. IR spectra were recorded on a JASCO FT/IR-4100 infrared spectrophotometer. The NMR spectra were recorded on a Varian 400MR FT-NMR (or Varian Unity INOVA500 FT-NMR) instrument at 400 MHz (or 500 MHz) for ^1^H and 100 MHz (or 125 MHz) for ^13^C in CDCl_3_. ESIMS data were obtained with a Finnigan LCQ ion-trap mass spectrometer. HRESIMS data were recorded on a LTQ Orbitrap XL mass spectrometer. Silica gel (Merck, 230–400 mesh) was used for column chromatography. Precoated silica gel plates (Merck, Kieselgel 60 F-254, 0.2 mm) were used for analytical TLC. High-performance liquid chromatography was performed on a Hitachi L-2455 HPLC apparatus with a Supelco C18 column (250 × 21.2 mm, 5 μm).

### 3.2. Animal Material

Soft coral *P. thyrsoide* was collected by hand using SCUBA off the coast of Orchid Island, located off Taiwan’s southeastern coast, in August 2008, at a depth of 10–15 m, and stored in a freezer until extraction. A voucher sample was deposited at the Department of Marine Biotechnology and Resources, National Sun Yat-sen University.

### 3.3. Extraction and Separation

The frozen bodies of *P. thyrsoide* (3.1 kg, wet wt) were sliced and exhaustively extracted with dichloromethane (1 × 10 L). The EtOAc extract (30.4 g) was chromatographed over silica gel by column chromatography and eluting with EtOAc in *n*-hexane (0–100%, stepwise) then with acetone in EtOAc (50–100%, stepwise) to yield 30 fractions. Fraction 21, eluting with *n*-hexane–EtOAc (2:1), was further purified over silica gel using *n*-hexane–acetone (7:1) to afford six subfractions (A1–A6). Subfraction A2 was separated by reversed-phase HPLC using MeOH–H_2_O (4:1) to afford **4** (1.8 mg), **5** (0.6 mg), **6** (18.3 mg) and **7** (8.8 mg), and subfraction A4 was separated by reversed-phase HPLC using MeOH–H_2_O (2:1) to afford **1** (10.2 mg), **2** (14.6 mg), **3** (0.8 mg) and **8** (5.1 mg).

Paralemnolin Q (**1**): white solid; mp 118 °C; [α]^26^ _D_ +26 (*c* 0.24, CHCl_3_); IR (neat) ν_max_ 3429, 2963, 2931, 2877 and 1735 cm^−1; 13^C and ^1^H NMR data, see Tables 1 and 2; ESIMS *m/z* 273 [M + Na]^+^; HRESIMS *m/z* 273.1468 [M + Na]^+^ (calcd for C_15_H_22_O_3_Na, 273.1467).

Paralemnolin R (**2**): white solid; mp 120 °C; [α]^26^ _D_ −42 (*c* 1.46, CHCl_3_); UV (MeOH) λ_max_ 211 (log ɛ = 3.5); IR (neat) ν_max_ 3401, 2960, 2937 and 1654 cm^−1; 13^C and ^1^H NMR data, see Tables 1 and 2; ESIMS *m/z* 273 [M + Na]^+^; HRESIMS *m/z* 273.1465 [M + Na]^+^ (calcd for C_15_H_22_O_3_Na, 273.1467).

Paralemnolin S (**3**): colorless oil; [α]^24^ _D_ +83 (*c* 0.08, CHCl_3_); IR (neat) ν_max_ 2958, 2933, 2881, 1719 and 1676 cm^−1; 13^C and ^1^H NMR data, see Table 1; ESIMS *m/z* 251 [M + H]^+^; HRESIMS *m/z* 251.1634 [M + H]^+^ (calcd for C_15_H_23_O_3_, 251.1642).

Paralemnolin T (**4**): colorless oil; [α]^24^ _D_ −28 (*c* 0.18, CHCl_3_); IR (neat) ν_max_ 3361, 2933, 2881 and 1652 cm^−1; 13^C and ^1^H NMR data, see Table 1; ESIMS *m/z* 269 [M + H]^+^; HRESIMS *m/z* 269.1742 [M + H]^+^ (calcd for C_15_H_25_O_4_, 269.1747).

Paralemnolin U (**5**): colorless oil; [α]^24^ _D_ −140 (*c* 0.06, CHCl_3_); IR (neat) ν_max_ 3365, 2937, 2877 and 1652 cm^−1; 13^C and ^1^H NMR data, see Table 1; ESIMS *m/z* 269 [M + H]^+^; HRESIMS *m/z* 269.1746 [M + H]^+^ (calcd for C_15_H_25_O_4_, 269.1747).

Preparation of (*S*)- and (*R*)-MTPA esters of **1**. To a solution of **1** (1.0 mg) in pyridine (0.1 mL) was added (*R*)**-**MTPA chloride (10 μL), and the mixture was allowed to stand for 12 h at room temperature. After the evaporation of the solvent, the residue was subjected to short silica gel column chromatography using *n*-hexane–acetone (6:1) to yield the (*S*)-MTPA ester, **1a** (0.4 mg). The same procedure was applied to obtain the (*R*)-MTPA ester **1b** (0.9 mg) from the reaction of (*S*)-(+)-α-methoxy-α-(trifluoromethyl)phenylacetyl chloride with **1**. Selective ^1^H-NMR (CDCl_3_, 400 MHz) data of **1a**: δ 2.237 (1H, m, H-2a), 1.513 (1H, m, H-2b), 1.757 (1H, m, H-3a), 1.641 (1H, m, H-3b), 2.123 (1H, brs, H-6), 3.992 (1H, d, *J* = 6.4 Hz, H-8), 5.488 (1H, dd, *J* = 6.4, 2.0 Hz, H-9), 1.091 (3H, d, *J* = 6.8 Hz, H-13), 0.801 (3H, d, *J* = 6.8 Hz, H-14), 1.046 (3H, s, H-15); selective ^1^H NMR (CDCl_3_, 400 MHz) data of **1b**: δ 2.256 (1H, m, H-2a), 1.656 (1H, m, H-2b), 1.788 (1H, m, H-3a), 1.656 (1H, m, H-3b), 2.106 (1H, brs, H-6), 3.863 (1H, d, *J* = 6.8 Hz, H-8), 5.152 (1H, dd, *J* = 6.4, 2.0 Hz, H-9), 1.078 (3H, d, *J* = 6.8 Hz, H-13), 0.804 (3H, d, *J* = 6.8 Hz, H-14), 1.038 (3H, s, H-15).

### 3.4. Cytotoxicity Testing

Cell lines were purchased from the American Type Culture Collection (ATCC). Cytotoxicity assays of compounds **1**–**8** were performed using the Alamar Blue assay [19,20].

### 3.5. Neuroprotective Activity Assay

The method for neuroprotective assay was modified from previous study [18]. The human neuroblastoma SH-SY5Y cell line was cultured on 96-well plates. Compounds **1**–**8** were added to the cells 1 h before 20 μM 6-hydroxydopamine (6-OHDA) challenge. After 15 h incubation, 10 μL of Alamar Blue (Biosource, CA, USA) was aseptically added. The percentages of neuroprotection in 6-OHDA alone and control (without 6-OHDA and test compounds) groups were defined as 0% and 100%, respectively.

## 4. Conclusions

Our present investigation demonstrated that the Formosan soft coral *Paralemnalia thyrsoides* is a good source of bioactive substances. In our investigation of new and bioactive metabolites from the Formosan soft corals, this is the first study of *P. thyrsoides* collected from Orchid Island. From the neurological activity results, compounds **1**, **3**, **6** and **7**, in particular **6**, deserve further study for therapeutic potential against neurodegenerative diseases.

## Supplementary Material



## Figures and Tables

**Table 1:** ^13^C NMR data for compounds **1**–**5**.

**Position**	**1**, a δ_C_, mult.	**2**, b δ_C_, mult.	**3**, a δ_C_, mult.	**4**, a δ_C_, mult.	**5**, a δ_C_, mult.
1	69.5, CH c	73.1, CH	59.0, CH	118.5, CH	121.7, CH
2	36.4, CH_2_	29.4, CH_2_	25.5, CH_2_	77.8, CH	80.8, CH
3	28.8, CH_2_	24.1, CH_2_	23.3, CH_2_	30.5, CH_2_	31.1, CH_2_
4	36.3, CH	30.0, CH	32.5, CH	29.2, CH	33.7, CH
5	48.7, C	41.0, C	37.6, C	41.0, C	40.9, C
6	60.6, CH	56.9, CH	61.6, CH	58.9, CH	59.1, CH
7	211.6, C	201.8, C	210.6, C	107.3, C	107.5, C
8	73.7, CH	133.9, CH	40.3, CH_2_	33.2, CH_2_	33.4, CH_2_
9	111.3, CH	143.6, CH	78.2, CH	27.6, CH_2_	27.4, CH_2_
10	156.6, C	74.9, C	62.8, C	149.7, C	145.2, C
11	35.3, CH	26.4, CH	32.0, CH	37.1, CH	37.2, CH
12	61.9, CH_2_	64.6, CH_2_	67.6, CH_2_	72.2, CH_2_	72.2, CH_2_
13	18.8, CH_3_	14.9, CH_3_	17.2, CH_3_	18.7, CH_3_	18.6, CH_3_
14	14.2, CH_3_	13.8, CH_3_	14.5, CH_3_	15.9, CH_3_	16.3, CH_3_
15	19.5, CH_3_	17.6, CH_3_	17.7, CH_3_	19.7, CH_3_	21.1, CH_3_

a Spectrum recorded at 100 MHz in CDCl_3_;

b 125 MHz in CDCl_3_;

c Attached protons deduced by DEPT experiment.

**Table 2:** ^1^H-NMR spectral data for compounds **1**–**5**.

**Position**	**1**, a δ_H_ (*J* in Hz) c	**2**, b δ_H_ (*J* in Hz)	**3**, a δ_H_ (*J* in Hz)	**4**, a δ_H_ (*J* in Hz)	**5**, a δ_H_ (*J* in Hz)
1	4.22, dd (12.0, 4.8) c	3.81, brs	3.31, brs	5.57, dd (4.0, 1.6)	5.60, brs
2	2.19, dddd (14.0, 4.8, 2.4, 2.4)	2.18, dddd (15.0, 15.0, 5.0, 3.5)	2.19, dddd (15.6, 4.8, 2.4, 2.4)	4.37, t (4.0)	4.59, t (7.2)
	1.48, m	1.65, m	1.92, dddd (15.6, 12.0, 6.0, 1.6)		
3	1.74, ddd (14.0, 4.0, 4.0, 4.0)	1.70, m	1.37, m	1.93, m	1.84, m
	1.57, m	1.37, m	1.24, m	1.57, m	1.56, m
4	2.02, ddq (12.0, 4.0, 6.4)	2.55, ddq (15.5, 3.5, 7.0)	1.73, ddq (12.4, 2.8, 6.8)	2.02, ddq (12.8, 3.2, 6.8)	1.95, m
6	2.10, brs	2.25, d (5.0)	2.40, m	1.82, m	1.80, m
8	4.10, d (6.4)	6.39, s	2.80, dd (19.2, 2.8)	1.94, m	1.95, m
			2.64, dd (19.2, 2.8)	1.82, m	1.79, m
9	5.77, dd (6.4, 2.4)	6.39, s	3.53, t (2.8)	2.44, m; 2.30, m	2.46, m; 2.26, m
11	2.39, ddq (3.6, 3.2, 7.2)	2.36, m	2.38, m	1.92, m	1.91, m
12	4.18, dd (12.0, 3.2)	3.75, dd (12.5, 6.5)	3.81, dd (13.2, 4.8)	3.87, t (8.8)	3.88, t (8.8)
	3.38, dd (12.0, 1.2)	3.19, dd (12.5, 12.5)	3.31, dd (13.2, 9.6)	3.48, t (8.8)	3.49, t (8.8)
13	1.10, d (7.2)	0.70, d (7.0)	0.93, d (7.2)	1.10, d (5.2)	1.09, d (6.0)
14	0.79, d (6.4)	0.75, d (7.0)	0.76, d (6.8)	0.90, d (6.8)	0.92, d (6.8)
15	0.98, s	1.06, s	0.87, s	1.11, s	1.18, s

a Spectrum recorded at 400 MHz in CDCl_3_;

b 500 MHz in CDCl_3_;

c *J* values in Hz in parentheses.
