# Supplementary material for: Nardosinane-Type Sesquiterpenoids from the Formosan Soft Coral Paralemnalia thyrsoides
Source: Mar Drugs. 2011 Sep 16;9(9):1543–53. doi: 10.3390/md9091543 (PMC3225934; doi:10.3390/md9091543)
Supplement: Supplementary file 1 [file marinedrugs-09-01543-s001.pdf]

Nardosinane-type Sesquiterpenoids from the Formosan Soft Coral *Paralemnalia thyrsoides*

Chiung-Yao Huang,[1] Jui-Hsin Su, [1,2,3] Bo-Wei Chen,[1] Zhi-Hong Wen,[1] Chi-Hsin Hsu,[1] Chang-Feng Dai,[4] and Jyh-Horng Sheu [1,5 *]

[1] Department of Marine Biotechnology and Resources, National Sun Yat-sen University, Kaohsiung 804, Taiwan, R.O.C.

[2] National Museum of Marine Biology & Aquarium, Pingtung 944, Taiwan, R.O.C.

[3] Institute of Marine Biotechnology, National Dong Hwa University, Pingtung 944, Taiwan

[4] Institute of Oceanography, National Taiwan University, Taipei 112, Taiwan, R.O.C.

[5] Division of Marine Biotechnology, Asia-Pacific Ocean Research Center,
National Sun Yat-sen University, Kaohsiung, 804, Taiwan, R.O.C.

*To whom correspondence should be addressed. Tel.: 886-7-5252000 ext. 5030, Fax: 886-7-5255020. E-mail: sheu@mail.nsysu.edu.tw.

For compound 1:

Figure S1-1. ^{1}H NMR spectrum (400 MHz) of compound **1** in $CDCl_3$.

Figure S1-2. ^{13}C NMR spectrum (100 MHz) of compound **1** in $CDCl_3$.

Figure S1-3. ^{1}H NMR spectrum (400 MHz) of compound **1a** in $CDCl_3$.

Figure S1-4. ^{1}H NMR spectrum (400 MHz) of compound **1b** in $CDCl_3$.

For compound 2:

Figure S2-1. ^{1}H NMR spectrum (500 MHz) of compound **2** in $CDCl_3$.

Figure S2-2. ^{13}C NMR spectrum (125 MHz) of compound **2** in $CDCl_3$.

For compound 3:

Figure S3-1. ^{1}H NMR spectrum (400 MHz) of compound **3** in $CDCl_3$.

Figure S3-2. ^{13}C NMR spectrum (100 MHz) of compound **3** in $CDCl_3$.

For compound 4:

Figure S4-1. ^{1}H NMR spectrum (400 MHz) of compound **4** in $CDCl_3$.

Figure S4-2. ^{13}C NMR spectrum (100 MHz) of compound **4** in $CDCl_3$.

For compound 5:

Figure S5-1. ^{1}H NMR spectrum (400 MHz) of compound **5** in $CDCl_3$.

Figure S5-2. ^{13}C NMR spectrum (100 MHz) of compound **5** in $CDCl_3$.

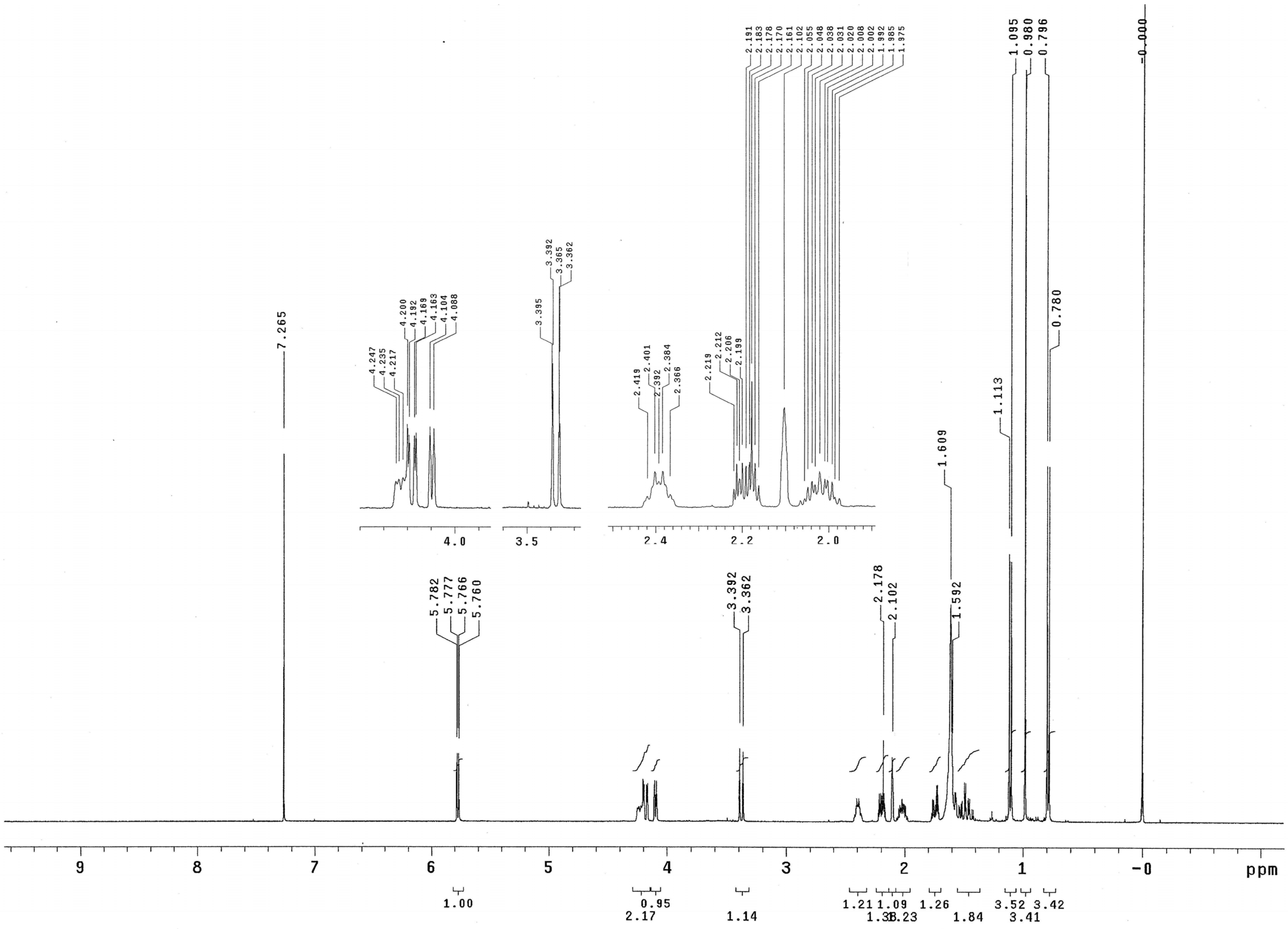

Figure S1-1. ^{1}H NMR spectrum (400 MHz) of compound **1** in $CDCl_3$.

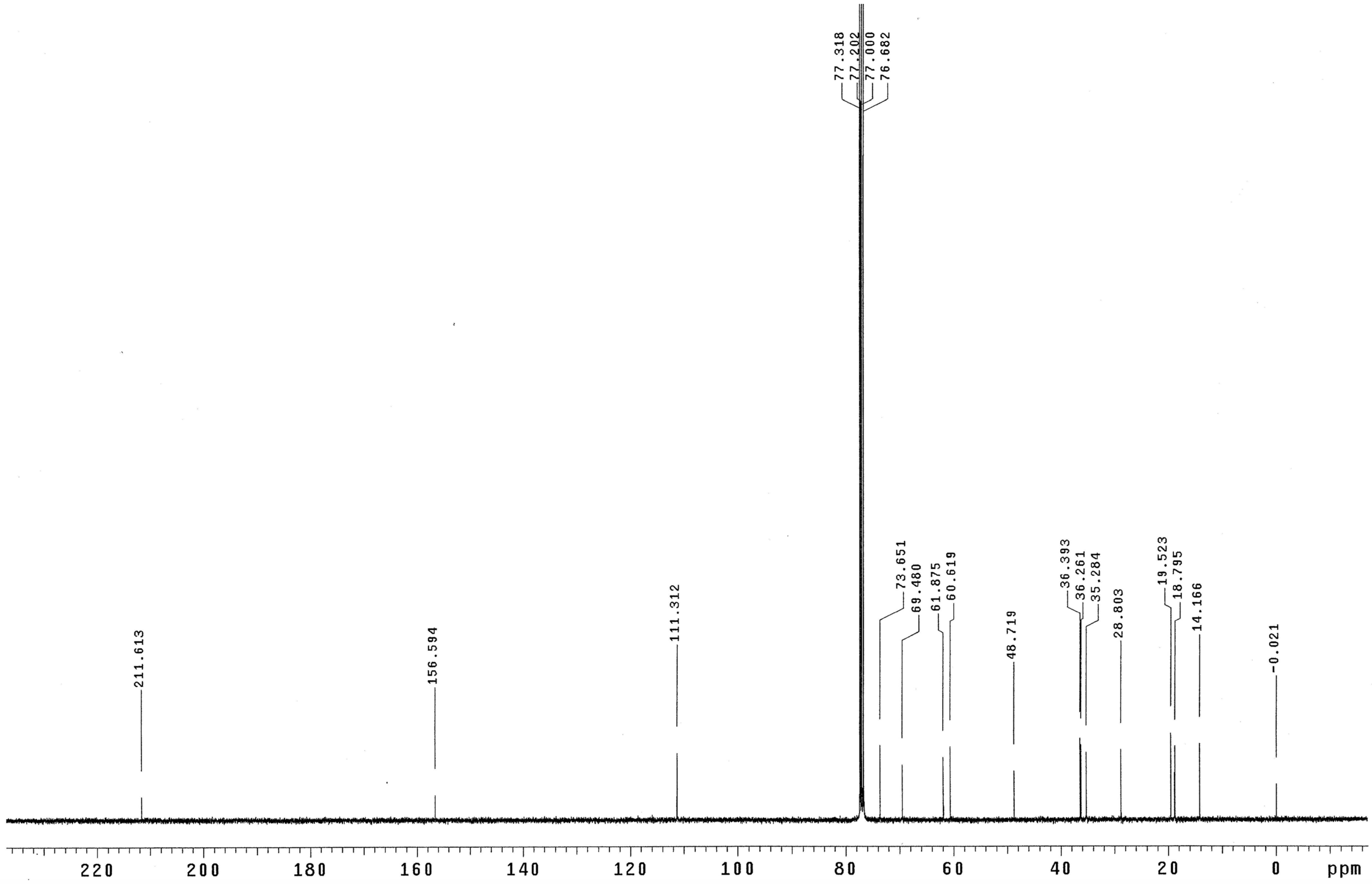

Figure S1-2. ^{13}C NMR spectrum (100 MHz) of compound **1** in $CDCl_3$.

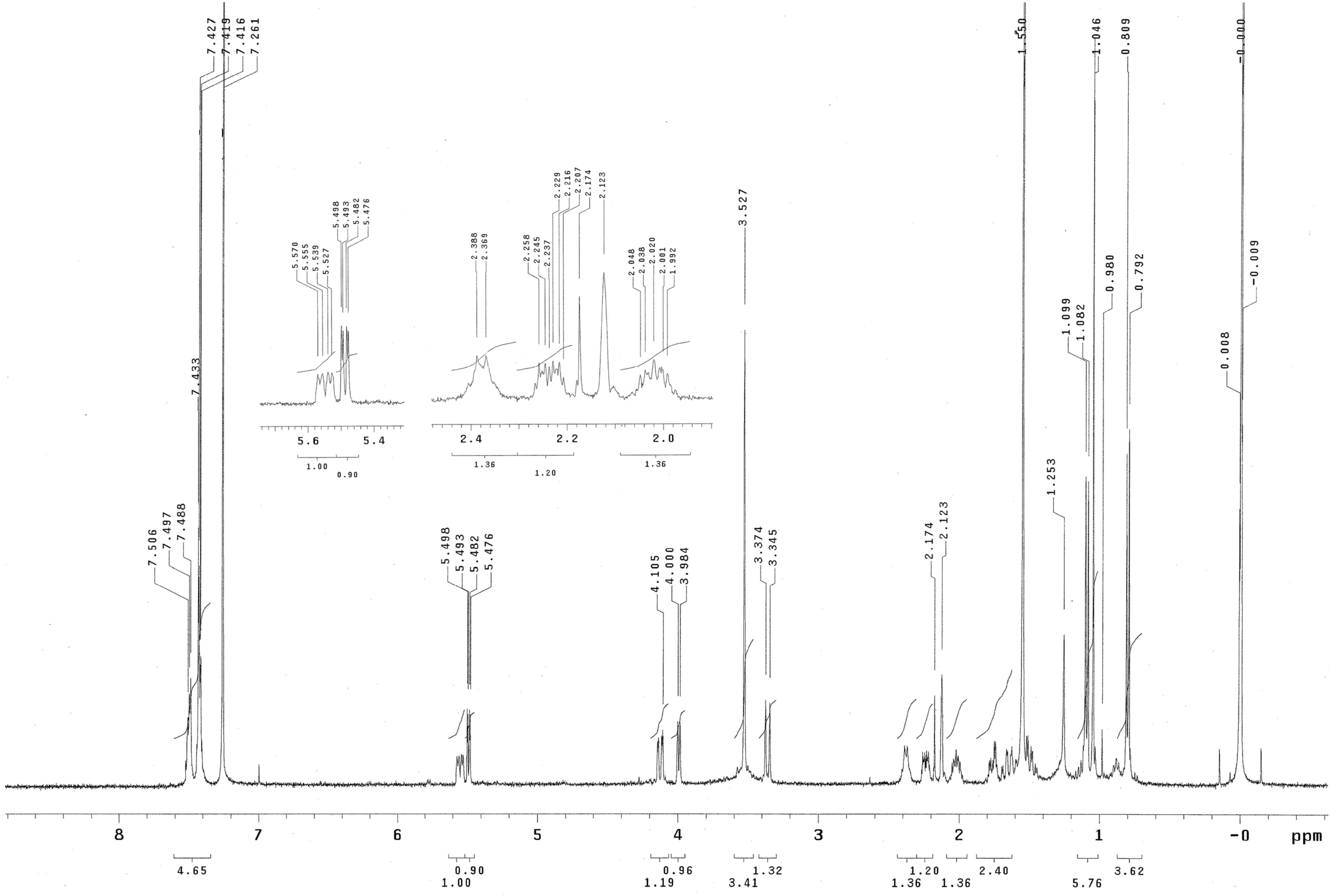

Figure S1-3. ^{1}H NMR spectrum (400 MHz) of compound **1a** in $CDCl_3$.

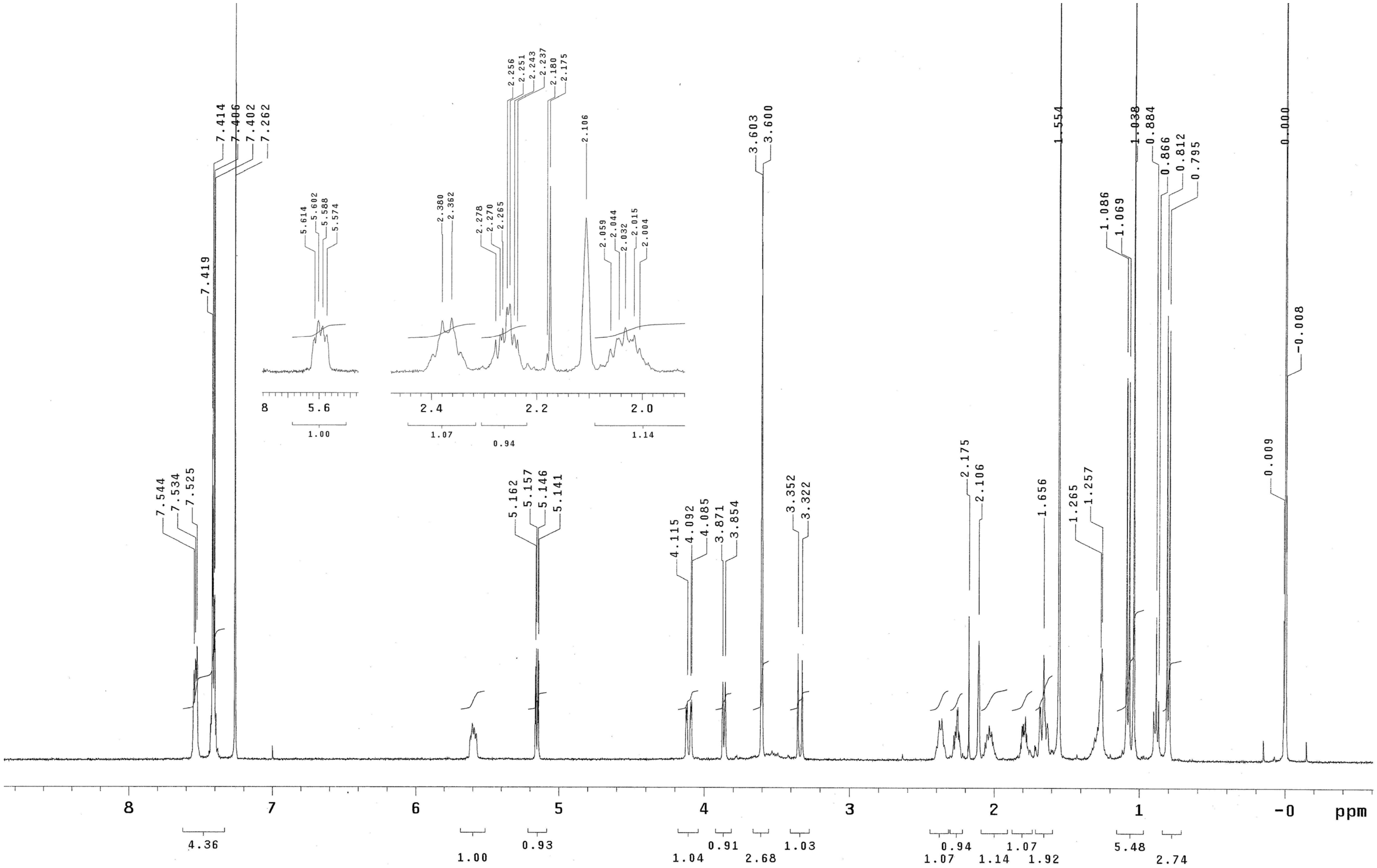

Figure S1-4. ^{1}H NMR spectrum (400 MHz) of compound **1b** in $CDCl_3$.

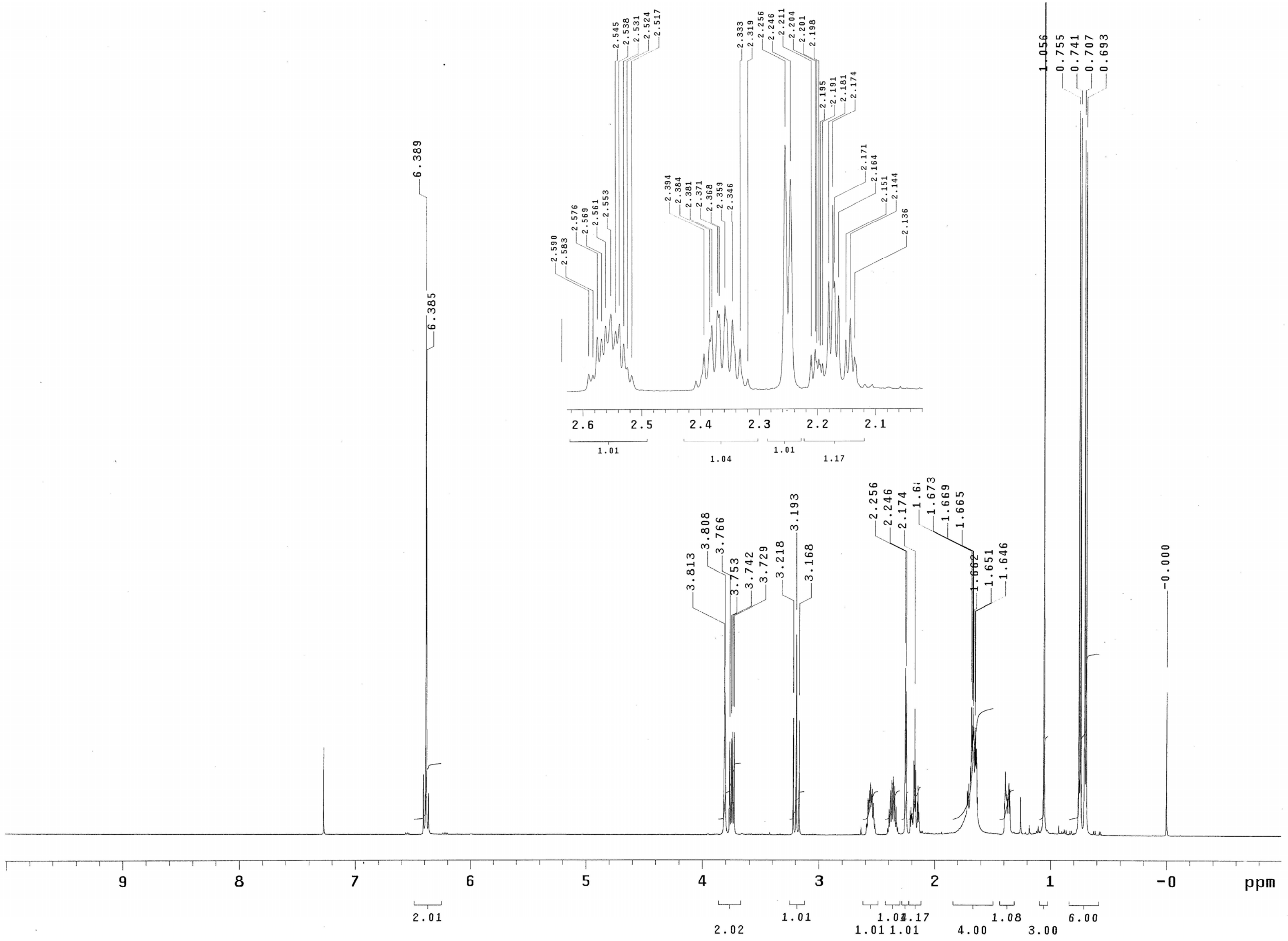

Figure S2-1. ^{1}H NMR spectrum (500 MHz) of compound **2** in $CDCl_3$.

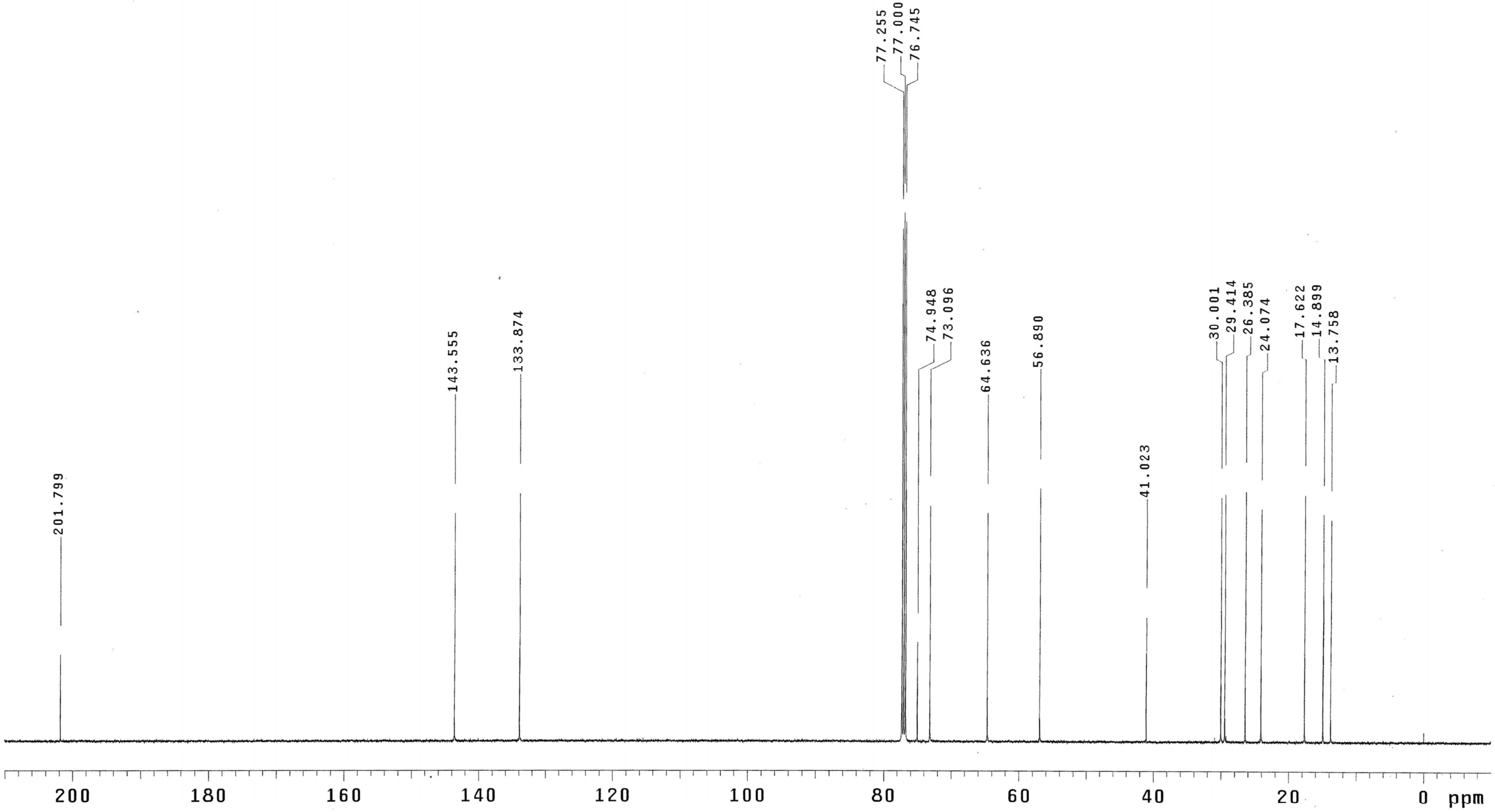

Figure S2-2. ^{13}C NMR spectrum (125 MHz) of compound **2** in $CDCl_3$.

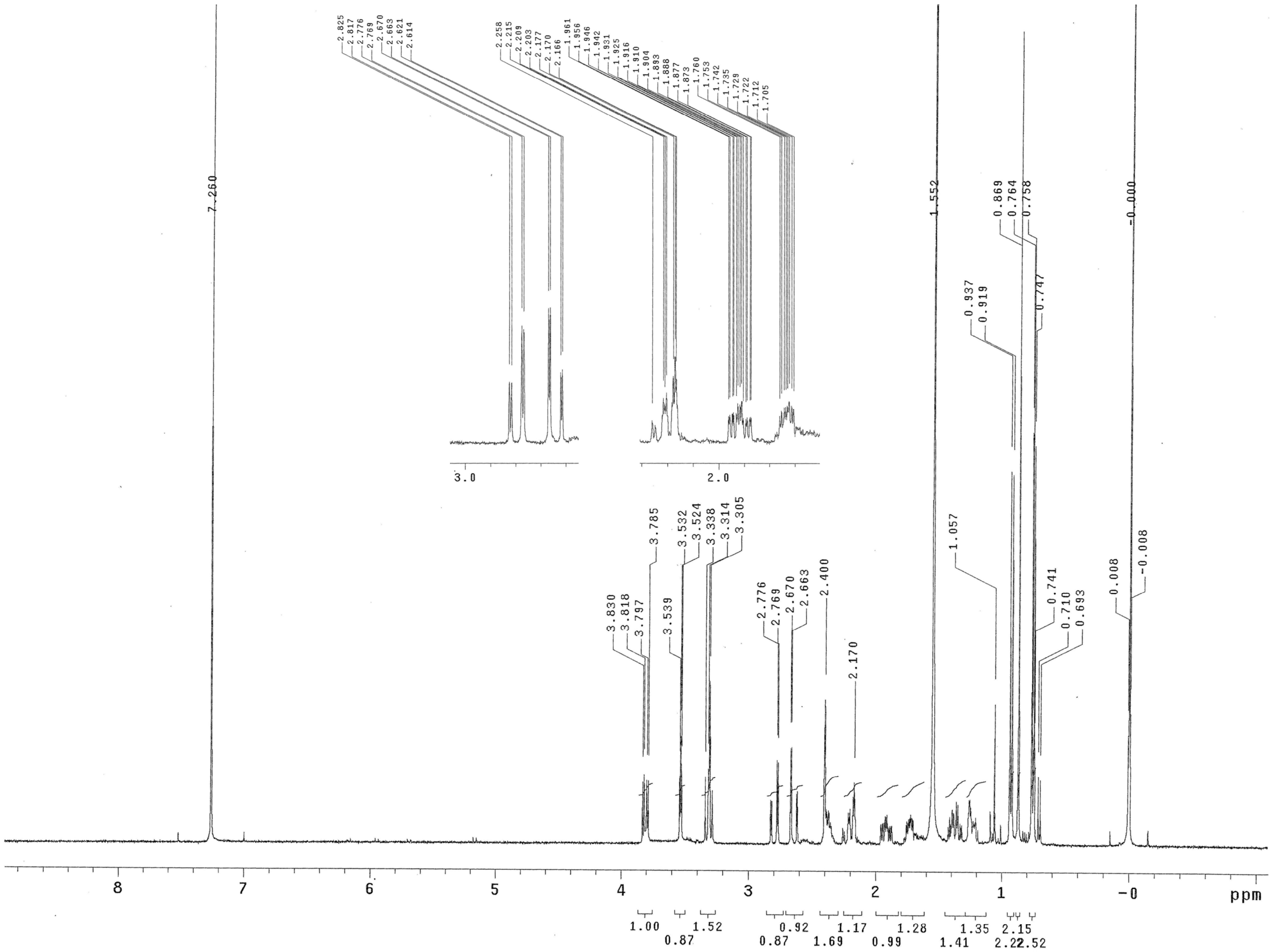

Figure S3-1. ^{1}H NMR spectrum (400 MHz) of compound **3** in $CDCl_3$.

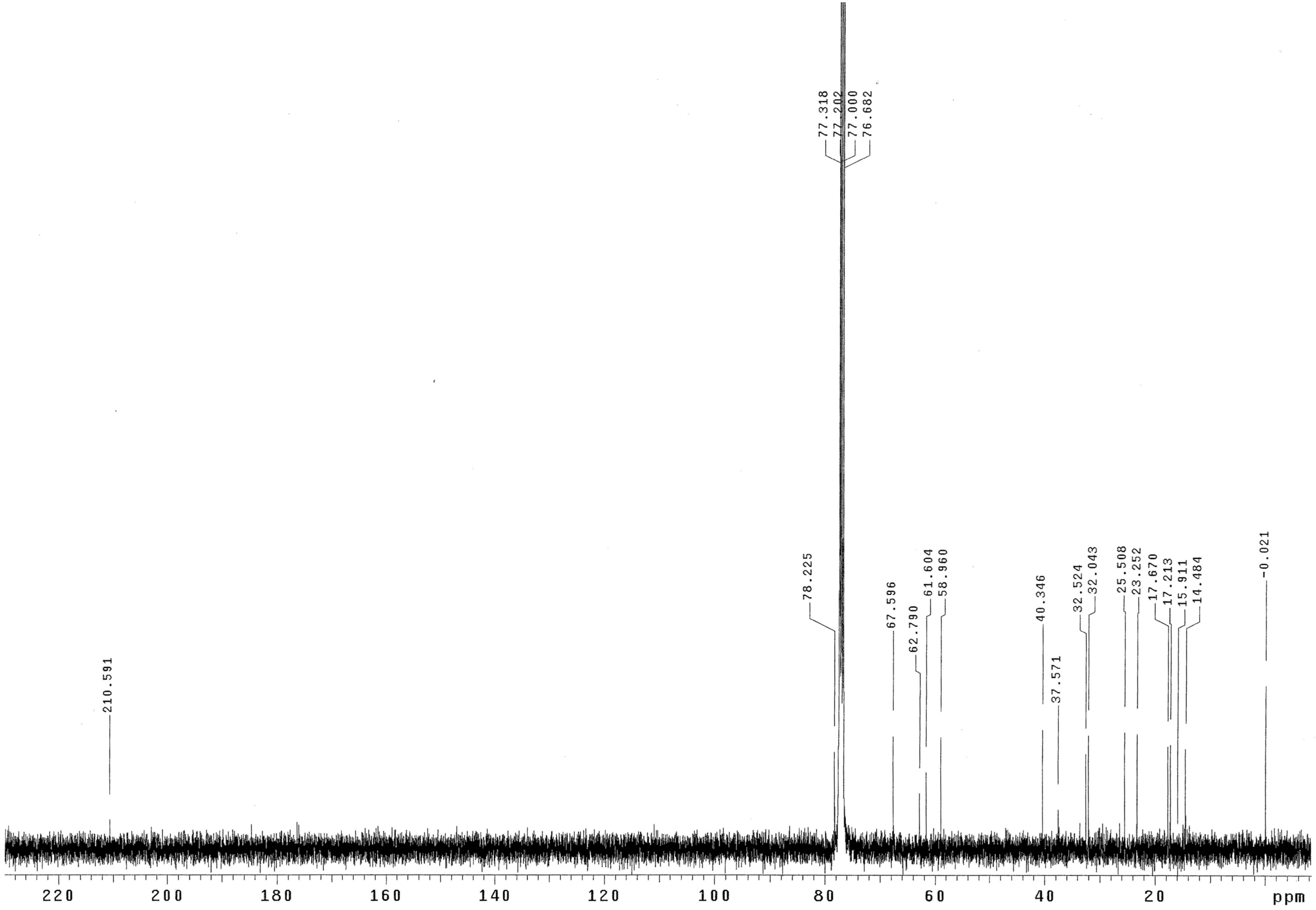

Figure S3-2. ^{13}C NMR spectrum (100 MHz) of compound **3** in $CDCl_3$.

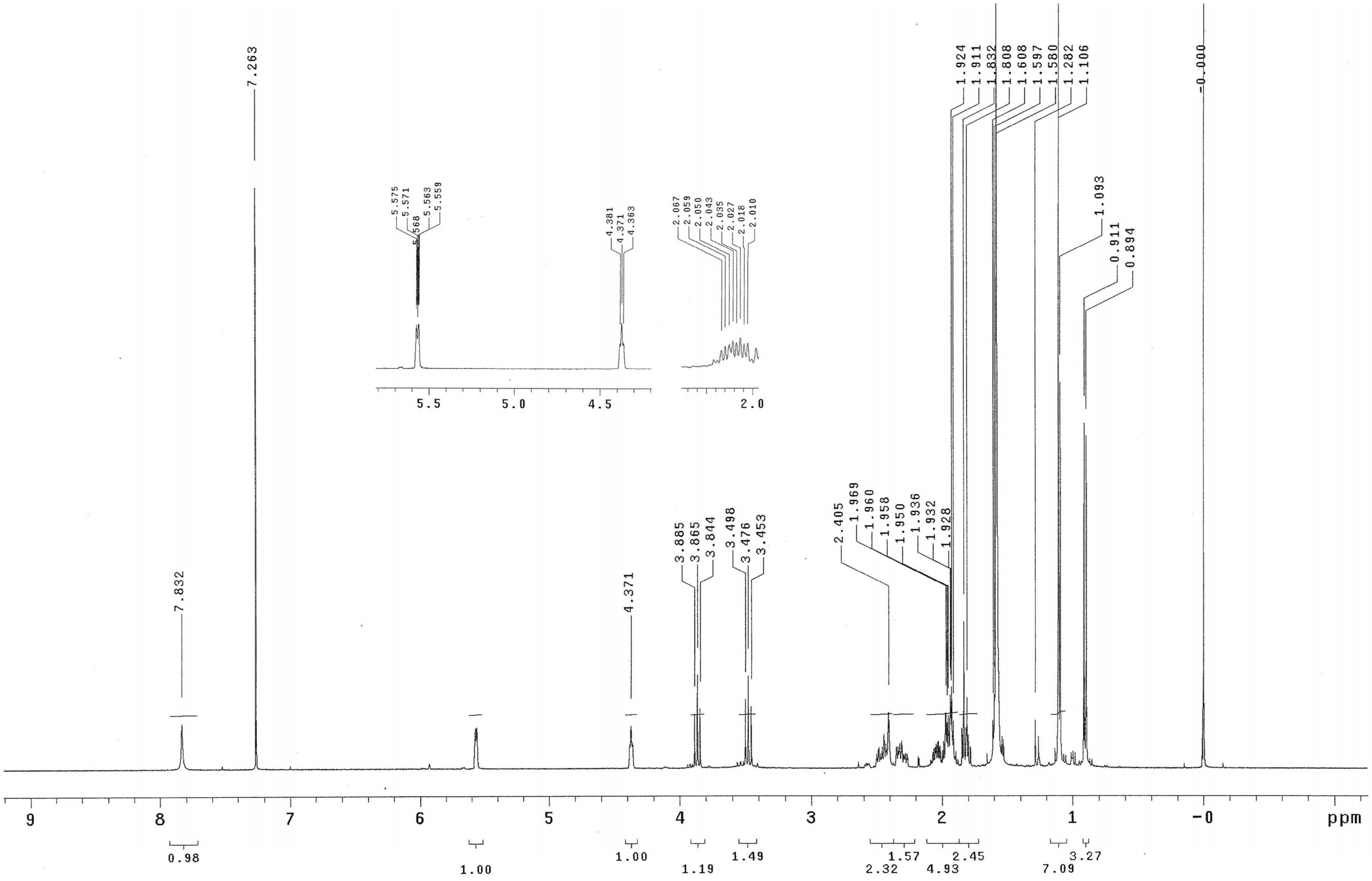

Figure S4-1. ^{1}H NMR spectrum (400 MHz) of compound **4** in $CDCl_3$.

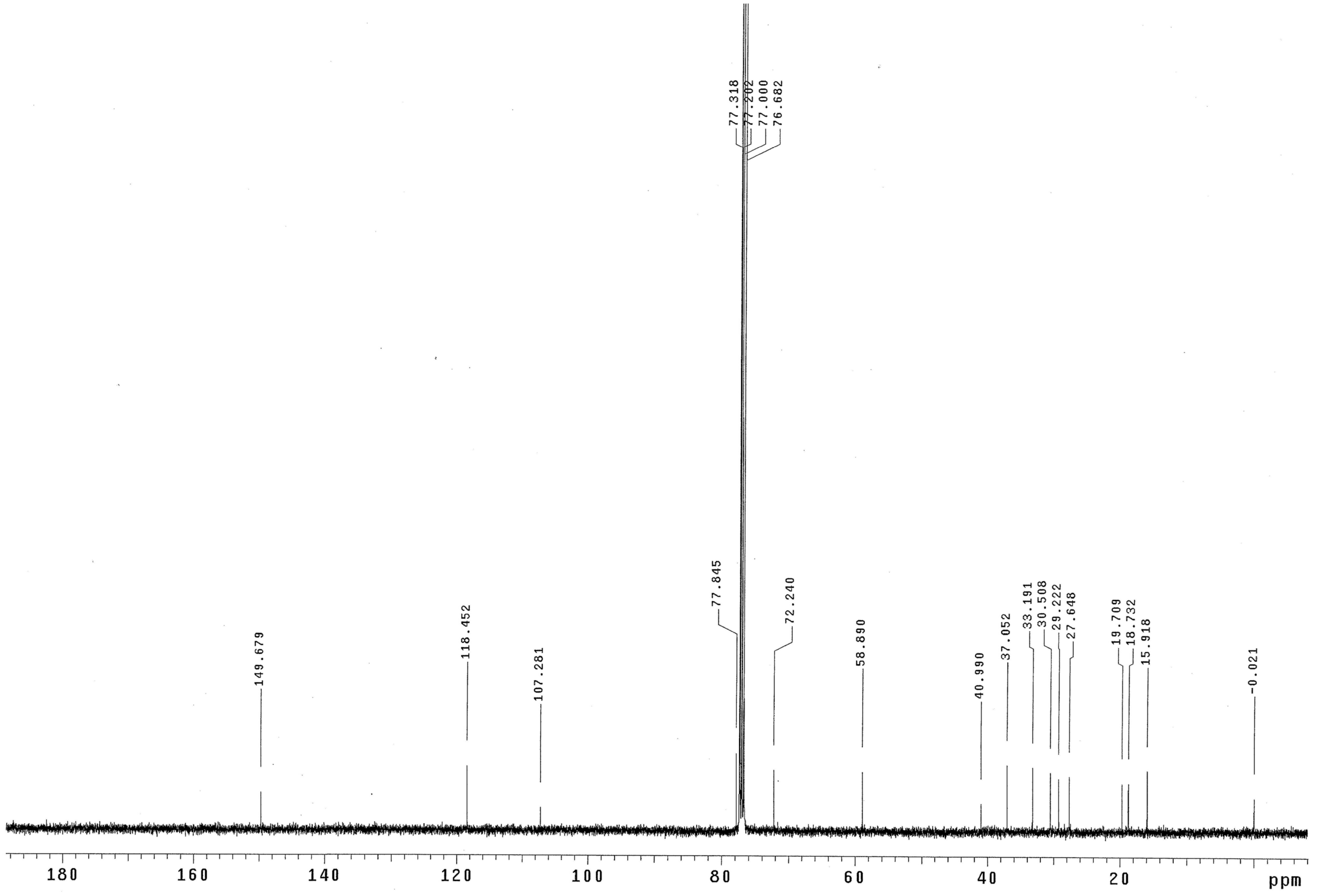

Figure S4-2. ^{13}C NMR spectrum (100 MHz) of compound **4** in $CDCl_3$.

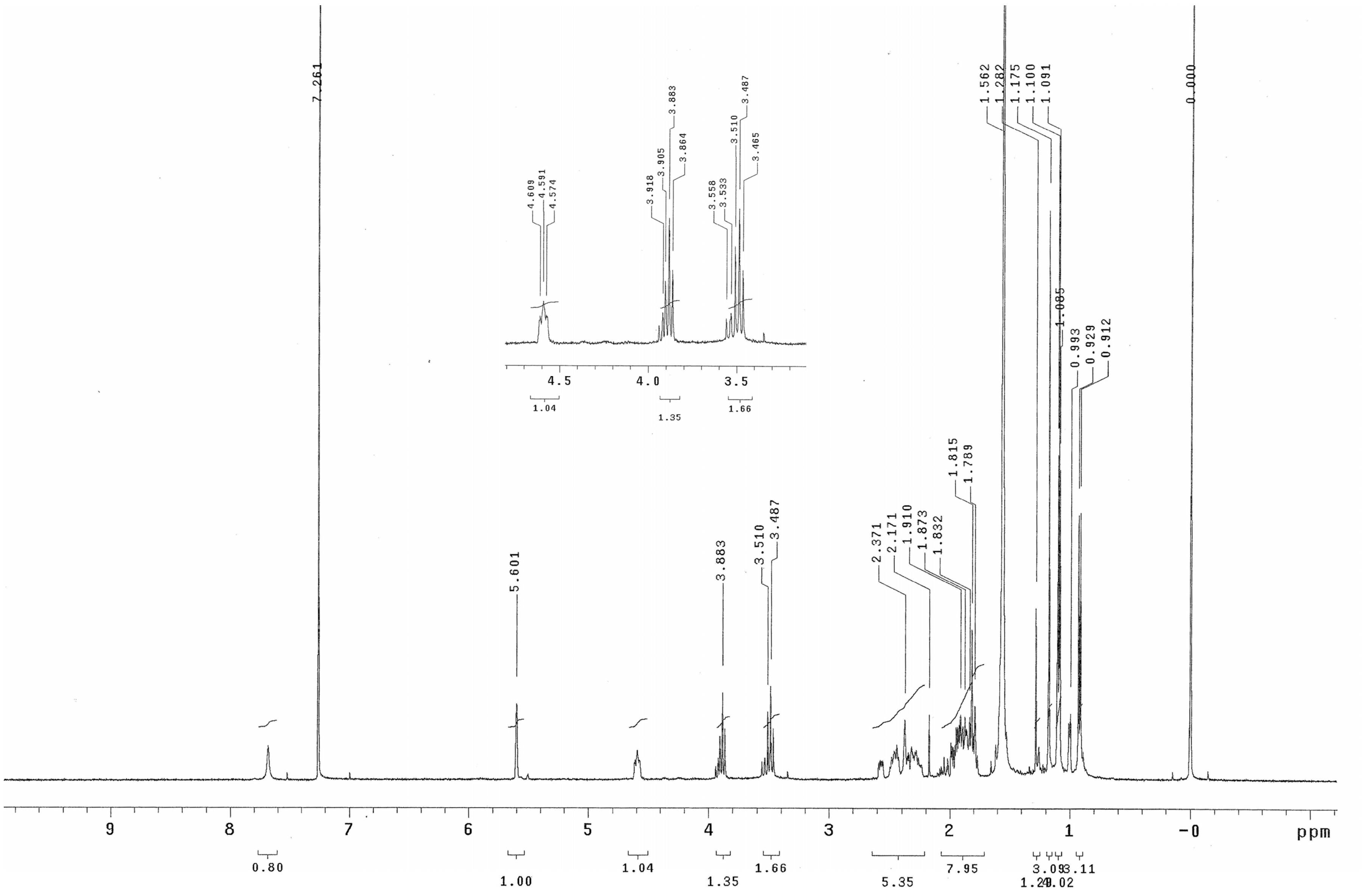

Figure S5-1. ^{1}H NMR spectrum (400 MHz) of compound **5** in $CDCl_3$.

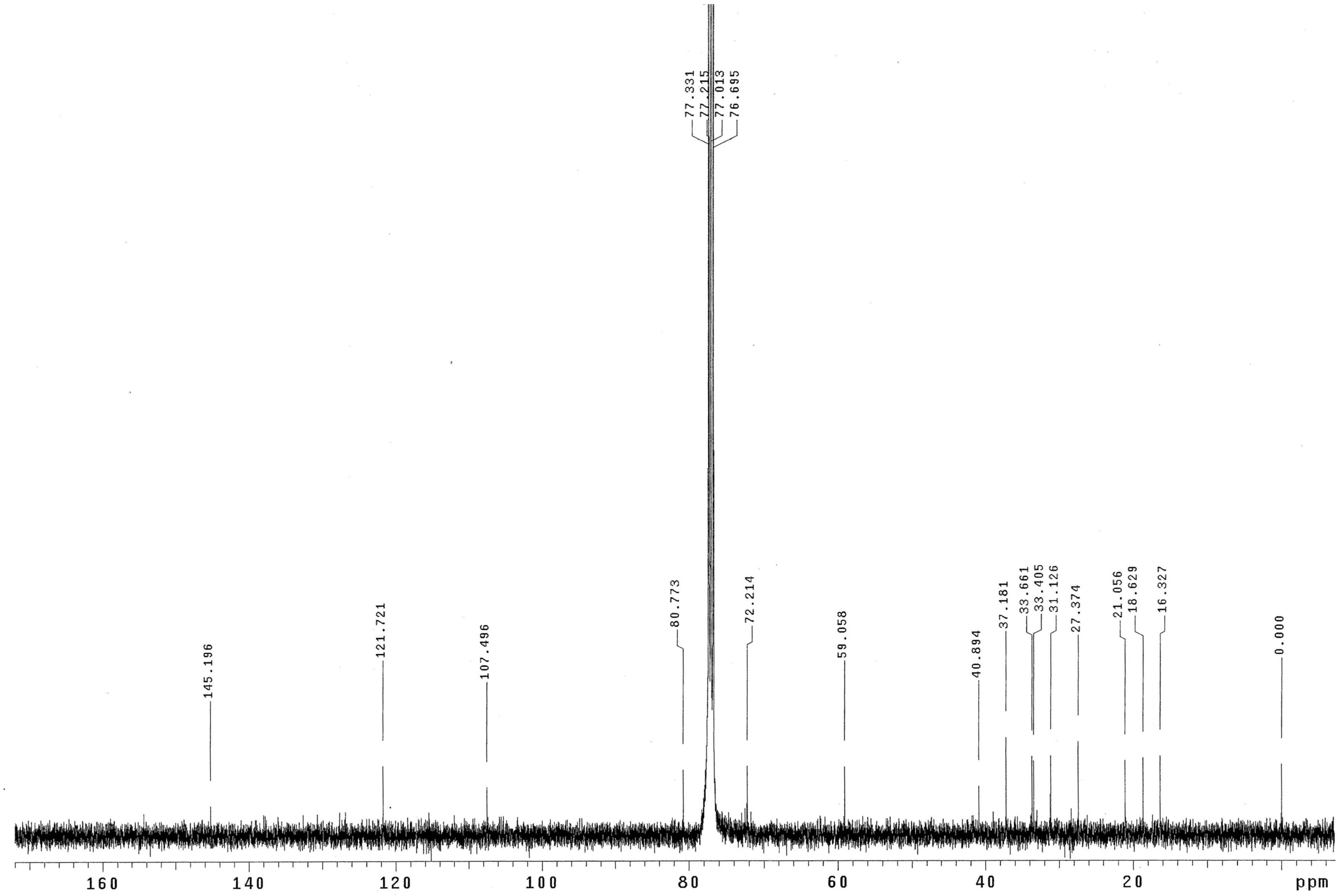

Figure S5-2. ^{13}C NMR spectrum (100 MHz) of compound **5** in $CDCl_3$.
